# Hyperglycemia and advanced glycation end products disrupt BBB and promote occludin and claudin-5 protein secretion on extracellular microvesicles

**DOI:** 10.1038/s41598-020-64349-x

**Published:** 2020-04-29

**Authors:** Slava Rom, Nathan A. Heldt, Sachin Gajghate, Alecia Seliga, Nancy L. Reichenbach, Yuri Persidsky

**Affiliations:** 1 Department of Pathology and Laboratory Medicine, Lewis Katz School of Medicine, Temple University, Philadelphia, PA 19140 USA; 2 Center for Substance Abuse Research, Lewis Katz School of Medicine, Temple University, Philadelphia, PA 19140 USA

**Keywords:** Blood-brain barrier, Diabetes

## Abstract

Cognitive impairment is a well-known complication of diabetes mellitus (DM). Microvascular compromise was described one DM complication. Recently we showed blood brain barrier (BBB) permeability and memory loss are associated with diminution of tight junctions (TJ) in brain endothelium and pericyte coverage and inflammation in cerebral microvessels and brain tissue paralleling hyperglycemia in mice of both DM types. The current study demonstrates that exposure of brain microvessels to hyperglycemic conditions or advanced glycation end products (AGEs) *ex vivo* resulted in significant abnormalities in membranous distribution of TJ proteins. We found significant increase in the amount of extracellular vesicles (EVs) isolated from DM mice and enhanced presence of TJ proteins, occludin and claudin-5, on EVs. Exposure of BMVECs to high glucose and AGEs led to significant augmentation of ICAM and VCAM expression, elevated leukocyte adhesion to and migration across BMVEC monolayers, and increased BBB permeability *in vitro*. Pericytes exposed to hyperglycemia and AGEs displayed diminished expression of integrin α1, PDGF-R1β and connexin-43. Our findings indicate BBB compromise in DM *ex vivo*, *in vitro* and *in vivo* models in association with BMVEC/pericyte dysfunction and inflammation. Prevention of BBB injury may be a new therapeutic approach to avert cognitive demise in DM.

## Introduction

In recent years, significant efforts have been made to define underlying causes of dementia (cognitive impairment) and its association with vascular pathology. A number of mechanisms have been suggested, including genetic defects in Parkinson’s disease and Alzheimer’s disease (AD), metabolic abnormalities [Diabetes Mellitus (DM)], toxic effects of drugs and environmental factors, etc.), chronic neuroinflammatory conditions (autoimmune or infectious origin), hypertension, stroke and traumatic brain injury. Several of these conditions congregate on defects in the brain microvasculature^1^ and its coupling to neuronal functions causing cerebral hypo-perfusion and subsequent neuronal demise^2,3^ leading to vascular cognitive impairment and dementia (VCID). DM is a metabolic disease related either to the failure to generate insulin for proper utilization of glucose (type 1) or insulin resistance when the produced hormone is not able to interact with its receptors (type 2). Both DM types result in high glucose levels, enhanced generation of reactive oxygen species (ROS) and end organ injury stemming from microvascular abnormalities^4^. Our recent study^3^ presented a strong association between hyperglycemia, enhanced BBB permeability and cognitive dysfunction in animal models of both DM types. Hyperglycemic animals showed a pro-inflammatory phenotype both in brain microvessels (BMVs) and signs of neuroinflammation in brain tissue. We found that disruption of the BBB was associated with cognitive decline in animals, similar to recently demonstrated BBB dysfunction linked to memory impairment in DM patients^5^. Immunohistochemical evaluation showed a significant decrease in pericyte coverage in BMVs and an increase in ICAM-1 expression in DM mice^3^. DM patients are assumed to develop an abnormal endothelial phenotype due to the high levels of circulating inflammatory markers and ICAM-1^6,7^. Previously, reduction in pericyte presence has been shown during DM both in the blood retinal barrier and BBB^8^. Pericytes provide functional support to the brain endothelium and their loss leads to enhanced permeability and tissue injury in DM^9^ and neuroinflammatory conditions^10^.

Membrane-bound extracellular vesicles (EVs) (diameter ~30–400 nm) are important mediators of intercellular communication among different tissues and organs in a wide spectrum of biological functions^11^. EVs are detected in most bodily fluids, and contain proteins, RNA, and lipids and can be categorized into at least one of three groups (exosomes, microvesicles and apoptosis bodies). The first group is released via the fusion of multivesicular bodies with the plasma membrane. The second group is released by budding of the plasma membrane, and the third group of EVs is shed from dying cells^11^. While EVs have been studied in the context of many diseases, little is known about EVs in the context of human diabetes^12,13^ and their relationship to vascular pathology. Recent studies in the db/db DM type 2 mouse model have been shown that EVs isolated from adipose tissue can activate macrophages and promote inflammatory signals^13^. Other reports have shown that platelet-derived EVs expressed different patterns in expression and procoagulant activity in patients with both DM types^14^.

In this report, we show that EVs isolated from DM mice express high levels of TJ proteins, occludin and claudin-5. BMVs, subjected to hyperglycemic conditions or AGEs in *ex vivo* settings, exhibited abnormal occludin and claudin-5 membrane TJ localization. Using our *in vitro* model of BBB, utilizing primary human brain microvascular endothelial cells (BMVEC) and primary human pericytes, we demonstrate defective barrier function by transendothelial electrical resistance (TEER) in hyperglycemic conditions. BMVECs displayed increased expression of adhesion molecules such as VCAM and ICAM when exposed to high glucose (HG) or AGEs, which resulted in augmented leukocyte adhesion to and crossing of the endothelial layer. RhoA and Rac1 GTPases have shown a significant increase in their activation in BMVEC stimulated with HG and AGE treatments. Since RhoA and Rac1 are small GTPases that control cytoskeleton, TJ and adhesion molecule expression in BMVEC and endothelial cells^15–17^, their activation in DM environment might explain barrier dysfunction.

Expression of integrin α1 [a key molecule guaranteeing adhesion to basement membrane (BM) matrix on pericytes] was altered in hyperglycemic conditions *in vitro*. mRNAs of BM proteins such as fibronectin, nidogen were also found to be down-regulated in a hyperglycemic environment in pericytes. Pericytes supporting BBB function also displayed a decrease in expression of the crucial pericyte receptor, PDGF-Rβ, which assures proper pericyte function and barrier integrity^10^. Pericytes, along with brain endothelium, synthesize the BM and its abnormalities are known to be present in a number of neurodegenerative and neurovascular diseases^18^. This report offers evidence from *in vitro* models and *ex vivo* treated BMVs and DM serum-isolated EVs for the causes of BBB dysfunction, and might lead to development of future therapeutics to reduce its burden.

## Materials and methods

### Reagents

Glyoxal (GO) and methylglyoxal (mGO) were obtained from Santa Cruz Biotechnology (Santa Cruz, CA). Lipopolysaccharides from *Escherichia coli* O111:B4 (LPS) and streptozotocin (STZ) were from Sigma/Aldrich (St. Louis, MO). Monocyte chemotactic protein-1 (MCP-1) was from R&D Systems (Minneapolis, MN). Rho inhibitor CTO4 and Rac activator CN04 were from Cytoskeleton (Denver, CO). Human tumor necrosis factor alpha (TNFα) was from Peprotech (Rocky Hill, NJ). ROS inhibitor, Trolox, and caspase inhibitor, Z-VAD-FMK, were purchased from Selleck Chemicals (Houston, TX).

### Animals and induction of diabetes

C57BL/6 mice (10-week old male) were acquired from the Jackson Laboratory (Bar Harbor, ME). To achieve statistical significance in each experiment, mice were divided into groups of 6 to 10 animals (exact numbers for each experiment are indicated in figure legends). All *in vivo* experiments were approved by the Temple University Institutional Animal Care and Use Committee in accordance with guidelines based on the National Institutes of Health (NIH) guide for care and use of laboratory animals and ARRIVE (Animal Research: Reporting *In Vivo* Experiments) guidelines (www.nc3rs.org.uk/arrive-guidelines). Diabetes type 1 was induced as described^3^. In short, C57BL/6 mice (25-30 g body weight) were randomly divided into groups. One group received once daily intraperitoneal (i.p.) injection of streptozotocin (STZ) for five consecutive days (50 mg/kg in citrate buffer, pH 4.5, freshly made every day). Control group mice received citrate buffer only. The first day of STZ injection was assigned as the starting time for diabetes. Serum glucose concentrations were monitored on 7 days, 4, 8 and 12 weeks after the start. Blood glucose levels (BGL) were determined by glucose analyzer (Bayer Contour, Ascensia Diabetes Care, Parsippany, NJ), according to manufacturer’s instructions.

### Brain microvessel isolation and *ex vivo* treatment

Mouse brain microvessels (BMVs) were isolated using a modified protocol based on previously published studies^19–21^. In short, mice were overdosed with CO_2_ and their brains harvested. All following steps were carried out on ice (or at 4 °C). Following a wash in phosphate-buffered saline, the brains were homogenized using a Dounce homogenizer (0.25 mm clearance) (whole brain is defined as the S0 fraction; the nomenclature of S0, S1, and S5 describes the BMVs fractionation steps consistent with the BMVs isolation procedure as described below and previously by Yousif^20^. Overall, 15 mL of 30% Ficoll was added to 10 mL of the homogenate and mixed thoroughly. The resulting density gradient was centrifuged at 5,800 × g for 20 minutes (the pellet is defined as the S1 fraction). The pellet was resuspended in 1 mL phosphate-buffered saline with 1% bovine serum albumin and passed through a glass bead column, with 100 μm nylon mesh filter on the top and a 40-μm nylon mesh filter at the bottom. The glass beads were gently agitated in phosphate-buffered saline with 1% bovine serum albumin to obtain BMVs. The resulting sample (defined as the S5 fraction) was washed with bovine serum albumin-free phosphate-buffered saline and resuspended in complete RPMI media with 10% fetal bovine serum and 1% penicillin-streptomycin. BMVs were deposited on 8-well chamber slides (Thermo Fisher, Waltham, MA) which had been coated with 0.01% poly-L-lysine (Sigma/Aldrich) and allowed to settle for 1 hour at 37 °C prior to addition of treatments for 48 hours at the concentrations shown.

### Immunocytochemistry

At conclusion of *ex vivo* treatment, BMVs were fixed for 10 minutes at 25 °C by 4% formaldehyde. After 3-4 PBS washes, BMVs were permeabilized with 0.1% Triton X-100 in PBS for 5 minutes, then washed 3-4X in PBS and blocked in 5% BSA for 30 minutes. Incubation with primary antibodies for either claudin-5 (1:25, Cat 35-2500, Invitrogen, Carlsbad, CA) or occludin (1:25, Cat 71-1500, Invitrogen) occurred overnight at 4 °C. Following 3-4 washes in 25 °C PBS, a 5% BSA block was performed for 30 minutes and incubation with AlexaFluor 488-conjugated secondary antibodies for either goat α-mouse (1:500, A28175, Thermo Fisher) or goat α-rabbit (1:500, A11034, Thermo Fisher) was performed for 45 minutes. BMVs were then incubated with DyLight 594 labelled *Lycopersicon esculentum* lectin (1:200, DL-1177, Vector Laboratories, Burlingame, CA) for 1 hour and washed 3-4 X with PBS. A solution of DAPI (1:2500) in PBS was added for 30 minutes and then 4-5 washes in PBS were performed before coverslip mounting with ProLong Gold Antifade reagent (ThermoFisher). Microscopic examination of brains used a standardized protocol with sections from the neocortex (frontal and parietal), basal ganglia, hippocampus, midbrain, pons, medulla, and cerebellum. Paraffin sections (5 *μ*m) were stained with hematoxylin-eosin. One section from the frontal cortical lobe was used for the evaluation of neuroinflammation and BBB structure by immunohistochemistry using ZO-1 antibodies (1:200, clone ZMD.437, ThermoFisher). Primary antibodies were detected by Dako Envision Kit (Dako, Carpinteria, CA) with DAB chromogen. Samples were imaged under 400x objective magnification using a DS-Fi1c camera (Nikon Corporation, Tokyo) configured to an upright microscope (Eclipse 55i, Nikon). For each animal, 4–6 randomly selected fields in the cortex and basal ganglia were analyzed, as described^3^. Immunostaining for ZO-1 was evaluated in blinded fashion using semi-quantitative scoring: 0 – no staining, 1 – weak staining (<10% of capillaries), 2 – moderate, variable labeling (10-75% capillaries), 3 – strong, variable staining (>75% capillaries) and 4 – strong, uniform staining (95%).

### Imaging and analysis

All BMVs images were captured on a Nikon A1R confocal microscope using a 60x oil-immersion lens and NIS-Elements software (Nikon, Tokyo, Japan). Z-series images were acquired through the full depth of 7 vessels in each well. Gain and laser settings were adjusted for the antigen being imaged (claudin-5, occludin) and then kept constant across imaging of all treatment groups. The 3D Deconvolution tool was applied to all images and analysis of tight junction fluorescent intensity was carried out using the NIS-Elements General Analysis 3 package. All representative images are maximum intensity projections of the entire z-series viewed across the XY plane and are shown with identical LUT settings applied across all treatment groups and include only original captured channels, unaltered by the analysis process described below. Due to a significant incidence of background fluorescence outside of the BMVs within claudin-5 and occludin channels, a binary rendering of the vessel was created from the lectin channel and utilized to crop out fluorescence occurring outside the vessel. The binary rendering was generated by using the Threshold3D module (range 451 to 65535, Smooth x5, Clean x2, Fill Holes ON) and then FilterObjects3D module to remove objects with a volume less than 20 µm^3^. The binary vessel rendering was then input to MaskImage module, generating a new channel which included only claudin-5 or occludin fluorescence within the vessel space. All subsequent analysis functions utilized this channel as an input. In order to identify regions where tight junctional (TJ) complexes were located, several image pre-processing modules were combined to boost the intensity of linear staining patterns versus those which were more punctate or diffuse. The resulting image was used to create and refine a binary rendering of the TJs, which was then applied to the original vessel-masked image to determine the intensity of fluorescence occurring within TJ complexes. Specifically, the Local Contrast (degree 100%, radius 0.50 µm), Auto Contrast (low 1%, high 0.1%), and Smooth (count 3) modules were applied in sequence prior to use of the Threshold3D module (range 23000 to 65535, Smooth x3, Clean x2) to create a binary rendering. FilterObjects3D module was applied to remove objects with volume less than 0.4 µm^3^ or elongation index less than 1.5. The rendering was skeletonized using Skeleton3D module, then Dilate3D (count 4) and Smooth3D (radius 0.250 µm) modules were sequentially applied (Supplemental Fig. 1). To determine the ratio of fluorescent intensity occurring within the TJ versus other cellular locations, the sum fluorescent intensity of TJ versus non-TJ regions were quantified and divided by volume of their respective regions to arrive at mean fluorescent intensity (MFI) for each region. The MFI within TJ was divided by non-TJ MFI to generate the ratio shown. Of note, the non-TJ intensity and volume were calculated indirectly through subtraction of TJ values from total vessel values (Supplemental Fig. 2). In order to account for background autofluorescence occurring within the vessel space, the fluorescence within vessels incubated with only secondary antibody was measured. The average background value was subtracted from the vessel-masked images across all treatment groups before fluorescent measurements were made.

**Figure 1:**
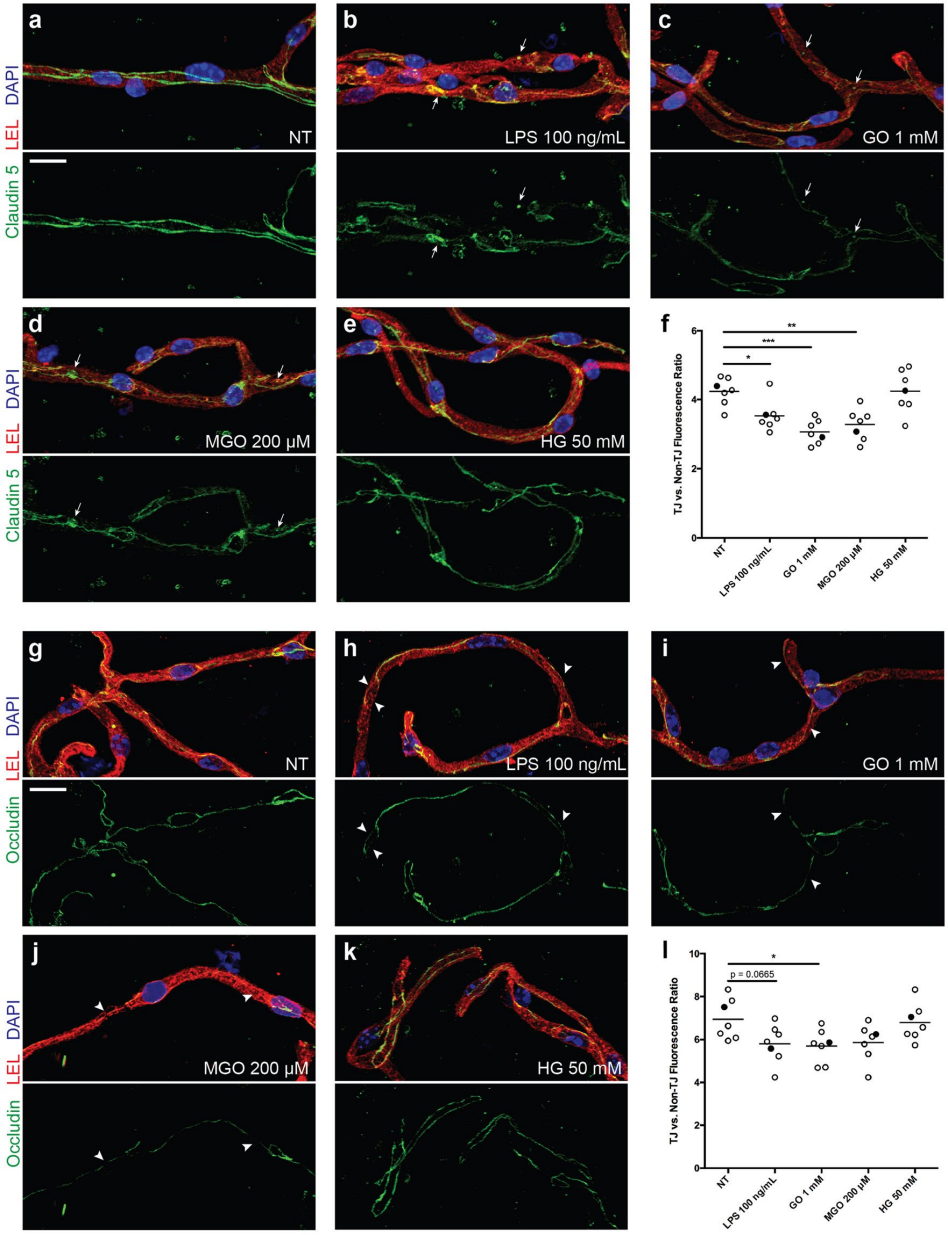
AGEs disrupt TJ complexes in microvessels treated *ex vivo*. Isolated vessels were labeled with *Lycopersicon esculentum* lectin (LEL, red), DAPI (blue), and immunostained for either claudin-5 (**a**–**f**) or occludin (**g**–**l**) (green). Multiple vessels (*n* = 7-8) were imaged from each treatment group and the ratio of fluorescent intensity for claudin-5 (**f**) or occludin (**l**) within versus outside of TJ complexes was quantified as described in methods. Filled circles indicate the datapoint corresponding to the representative image shown. Representative images were generated as a maximum intensity projection from the corresponding z-series across the XY plane. Arrows indicate points of punctate staining likely representing internalization of claudin-5. Arrowheads indicate localized regions of TJ with reduced occludin. Scale bar 10 μm, *p = 0.05, **p = 0.01, ***p = 0.001 versus NT. NT, no treatment; LPS, lipopolysaccharide; GO, glyoxal; MGO, methylglyoxal; HG, high glucose.

**Figure 2:**
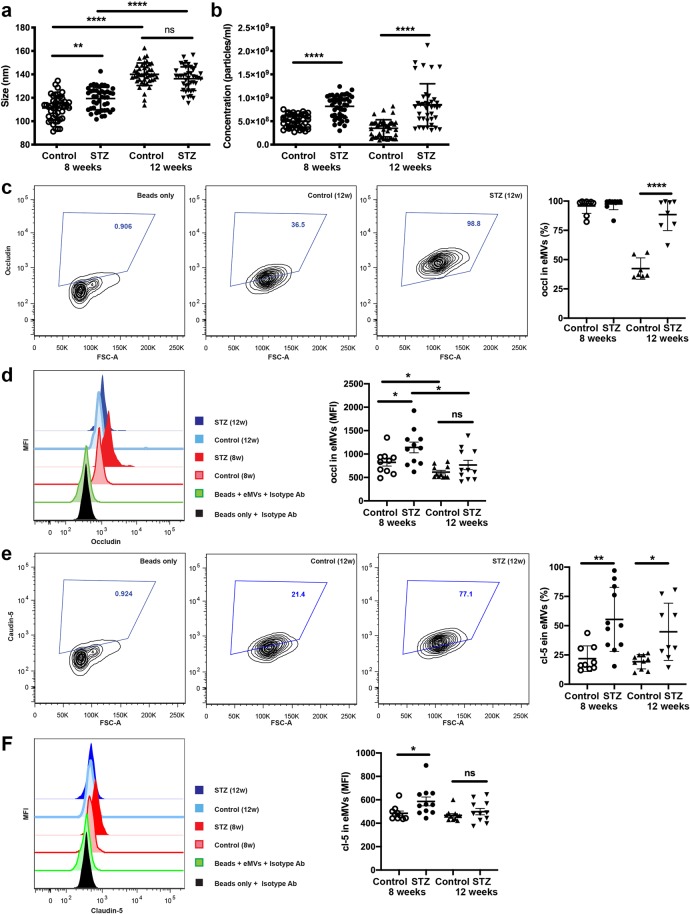
Hyperglycemic conditions result in an increased content of TJ proteins in extracellular microvesicles. EVs were isolated from the serum of STZ-treated or db/db mice or their controls. (**a**) NanoSight data on size and concentration of isolated EVs. Isolated EVs were stained with anti-occludin (**c**,**d**) and claudin-5 (**e**,**f**) antibodies. Flow cytometry analysis for percent of positive EVs (**c**,**e**) and mean fluorescent intensity (MFI) (**d**,**f**). Figure shows representative contour FACS plots as well as representative histograms to show changes in MFI. Data are presented as the mean ± SD. *p = 0.05, **p = 0.01, ****p = 0.0001.

### Cells

Primary BMVEC, isolated from vessels from brain resection tissue (showing no abnormalities) of patients undergoing surgery for treatment of intractable epilepsy, were supplied by Michael Bernas and Dr. Marlys Witte (University of Arizona, Tucson, AZ) and maintained as described^22,23^. Primary human monocytes were obtained from the University of Nebraska Medical Center Cell Core (Omaha, NE), maintained as described^24^, and used within 24 hr of isolation. Procedures were approved by the Institutional Review Board (IRB) of Temple University School of Medicine according to the ethical guidelines of the Helsinki Declaration of 1975 (and as revised in 1983). Primary human brain vascular pericytes were purchased from ScienCell Research Laboratories (Carlsbad, CA). We established pericyte culture conditions providing a quiescent, non-proliferating phenotype similar to ones present in the CNS under physiologic conditions. Use of growth medium containing 5% of growth factors slowed pericyte proliferation rate, with a majority of cells in the G1 phase of the cell cycle^25^. These culture conditions had no effect on pericyte viability (data not shown). Pericyte cultures were free of glial or endothelial cell contamination (Supplemental Material, Fig. 1). BBB models were assembled as previously described^24^ with BMVEC alone, pericytes alone, or BMVEC co-cultured over pericytes (5:1 seeding ratio of BMVEC:pericytes).

### Monocyte adhesion and transmigration assays

Adhesion assays were performed as described^21–23^. BMVEC were pretreated overnight with DM relevant stimuli, high glucose, glyoxal (GO) or methylglyoxal (mGO), followed by stimulation with TNFα (75 ng/ml) for 1 hr. Treatments were removed prior to monocyte introduction. Fluorescently-labeled monocytes (2.5 × 10^5^ cells/well) were added to the endothelial monolayers for 15 min at 37 °C. After adhesion, monolayers were washed and relative fluorescence of the attached monocytes was acquired on a fluorescence plate reader (Synergy 2 plate reader, BioTek, Winooski, VT). Results are presented as the mean ±SEM fold adhesion (number of adherent monocytes for each experimental condition divided by the basal adhesion of the untreated control). Transendothelial migration assays were done as previously described^24^. BMVEC or BMVEC/pericytes were plated on rat-tail collagen type I coated FluoroBlok tinted tissue culture plates (3 μm pores, BD Biosciences, Franklin Lakes, NJ) at a density of 2.5 × 10^4^ BMVEC/insert and 0.5 × 10^4^ pericytes/insert one week prior to use for migration assays. Medium was replaced, cell monolayers were washed and monocyte chemotactic protein type 1 (MCP-1/CCL2, 30 ng/ml, R&D Systems) was added to the lower chamber. Monocytes were labeled with calcein-AM as described for adhesion assays, washed, placed in the upper chamber and allowed to migrate for 2 hr at 37 °C. The number of migrated monocytes was determined with ImageJ software, version 1.43 (NIH, Bethesda, MD) and is expressed for each experimental condition as the mean of triplicate determinations calculated as the number of migrated monocytes divided by the number of migrated monocytes in untreated, no chemoattractant control. Data are presented as percent of input defined as the number of monocytes that migrated to the lower chamber over the total monocytes initially placed in the upper chamber x100.

### Transendothelial electrical resistance (TEER)

BMVEC alone or in co-culture with pericytes were plated on collagen type I coated 96W20idf electrode arrays (Applied Biophysics, Troy, NY) and were treated with 25 mM glucose or AGEs for 30 hr. To inhibit RhoA or Rac1 GTPase activity, cells were pretreated with specific inhibitors, 1 μg/ml CT04 (Cytoskeleton) or 75 μM NSC23766 (EMD-Millipore, Burlington, MA), respectively. To inhibit ROS or apoptosis, cells were pretreated with specific inhibitors, 25 μM Trolox or 100 μM Z-VAD-FMK (both from Selleck Chemicals), respectively. TEER measurements were performed using the 1600R ECIS System (Applied Biophysics, Troy, NY) as described^21–23^. The results are presented as the average percent change from baseline TEER (expressed as average ± SEM) from at least three independent experiments consisting of four to six replicates each.

### PCR array and qPCR

Total RNA was extracted from cells using the PARIS RNA Isolation Kit (Thermo Fisher). Total RNA (200 ng) was converted to cDNA using the RT² cDNA Synthesis Kit (Qiagen, Hilden, Germany). Specific primers and probes for nidogen, fibrinogen, PDGFRβ and Cx-43 genes were obtained from Thermo Fisher and analyses were executed using the QuantStudio S3 real-time PCR system (Thermo Fisher). Amplification was examined using the ΔΔCt method, using a web-based data investigation tool (SABiosciences/Qiagen) by normalization to housekeeping genes and fold-change calculated from the difference between experimental condition and untreated control, as described^26^. Data are presented from two independent experiments in triplicate for each gene. To eliminate biased data, two people performed analysis in blinded fashion.

### RhoA and Rac1 guanosine triphosphatase (GTPase) activity assay

RhoA and Rac1 GTPase activity was measured in cell lysates prepared from primary BMVEC after stimulation with high glucose or AGEs. For a positive control, cells were stimulated with the GTPase activator CN04 (1 μg/ml) (Cytoskeleton Inc, Denver, CO). To inhibit RhoA or Rac1 GTPase activity, cells were pretreated with specific inhibitors, 1 μg/ml CT04 (Cytoskeleton) or 75 μM NSC23766 (EMD-Millipore), respectively, as described^27^. To measure RhoA and Rac1 GTPase activity, G-LISA RhoA and G-LISA Rac1 Activation Assay kits (Cytoskeleton) were used according to the manufacturer’s instructions.

### ICAM, VCAM and Integrin-α1 expression quantification by flow cytometry

Analysis of surface expression of adhesion molecules was performed using the conjugated antibodies ICAM-1-APC and vascular cell adhesion molecule 1 (VCAM-1)-FITC (BD Biosciences). Integrin-α1 protein was stained with anti- Integrin-α1 Alexa-Fluor 405 (Novus Biologicals, LLC., Centennial CO). Following staining, data were acquired with a FACS BD Canto II flow cytometer (BD Biosciences) and analyzed with FlowJo software v9.9.6 (Tree Star, Inc., Ashland, OR). Data were collected from at least 20,000 events and repeated twice with BMVEC or pericytes each time from different donors. The mean fluorescent intensity (MFI) of stain was calculated in a cell population and presented as average ± SD.

### Extracellular vesicle isolation and staining

EVs were isolated from 50 μl of mouse serum utilizing Exo-Quick solution (System Biosciences LLC., (SBI), Palo Alto, CA), according to the manufacturer’s instructions. Size and concentration of the EVs were analyzed on the NanoSight NS300 (Malvern Instruments Ltd., Westborough, MA). Isolated EVs were captured with CD63 Exo-Flow Capturing Kit (SBI) and stained with anti-claudin-5 Alexa 488 or anti-occludin Alexa 488 (both from Thermo Fisher). Following staining, data were acquired with a FACS BD Canto II flow cytometer (BD Biosciences) and analyzed with FlowJo software v9.9.6 (Tree Star).

### Reactive oxygen species (ROS) measurement

BMVEC or pericytes were treated with diabetes conditions and Reactive Oxygen Species (ROS) measurement was performed utilizing the cell permeant reagent, 2′,7′–dichlorofluorescin diacetate (DCFDA, also known as H2DCFDA or DCFH-DA), a fluorogenic dye that measures hydroxyl, peroxyl and other ROS activity within the cell according to the manufacturer’s instructions (Abcam, Cambridge, MA). Fluorescence was measured by plate reader and ROS production is presented as arbitrary fluorescence units for triplicate measurements from at least two donors as mean ± SD.

### Statistical analysis

Results are expressed as the mean ± SEM of experiments conducted multiple times. Multiple group comparisons were performed by one-way analysis of variance with Dunnett’s posthoc tests, as described^21,26,27^. Statistical analyses were performed utilizing Prism v8.3.0 software (GraphPad Software Inc., La Jolla, CA). Differences were considered significant at P values <0.05.

## Results

### Hyperglycemia leads to disruption of TJ complexes in *ex vivo* treated cerebral microvessles

DM is a metabolic disorder characterized by hyperglycemia leading to end-organ injury in various organs due to microvascular compromise (cardiovascular disease, nephropathy and retinopathy) and inflammation. BBB breakdown has been suggested as one of the causes of dementia in DM and AD^28^. Recently we have shown in animal models of DM types 1 and 2 decrease BBB integrity and promote a pro-inflammatory phenotype of brain endothelium (resulting in low level inflammation) that further exacerbates barrier injury^3^. In spite of our assumption that BBB integrity in DM would be strongly associated with down-regulation in TJ proteins, we found that expression of occludin was prominently upregulated in isolated BMVs from animals with DM. We hypothesized that BBB integrity might be compromised due to improper folding or incorporation in cell membranes that may reflect a compensatory phenomenon in DM. To check this hypothesis, we subjected BMVs to DM conditions *ex vivo* and assessed TJ localization (membrane incorporation). BMVs were isolated from mice, as described^21^, plated on EZ slides (Millipore), treated *ex-vivo* with HG or AGEs and analyzed for expression of TJ complexes (Fig. 1). Isolated vessels were labeled with *Lycopersicon esculentum* lectin (LEL, red), DAPI (blue), and immunostained for either claudin-5 (a-f) or occludin (g-l) (green) to allow vessel imaging with a Nikon A1R confocal microscope (Nikon, Tokyo, Japan). To rigorously evaluate TJ expression, gain and laser settings were adjusted for the antigen being imaged (claudin-5, occludin) and then kept constant across imaging of all treatment groups. The 3D Deconvolution tool was applied to all images and analysis of tight junction fluorescent intensity was carried out using the NIS-Elements General Analysis 3 package (Nikon). All representative images are maximum intensity projections of the entire z-series viewed across the XY plane. To determine the ratio of fluorescent intensity occurring within the TJ versus other cellular locations, the sum fluorescent intensity of TJ versus non-TJ regions was quantified and divided by the volume of their respective regions to arrive at mean fluorescent intensity (MFI) for each region (Supplemental Fig. 2). The MFI within TJ was divided by non-TJ MFI to generate the ratio shown (Fig. 1). Treatment with glyoxal (GO) caused significant reduction ~23% and ~18% in TJ expression for claudin-5 and occludin, respectively (Fig. 1). Methylglyoxal (MGO) presence resulted in significant diminution of ~20% in TJ staining for claudin-5 (Fig. 1d,f). Similar reductions in TJ localization for both occludin and claudin-5 were noticed in LPS *ex vivo*-treated BMVs (Fig. 1b,f,h,l) suggesting commonality of the hyperglycemic DM environment and pro-inflammatory conditions (LPS).

### Diabetes leads to increased presence of TJ proteins in extracellular microvesicles

In recent years, several groups reported that EVs could play important roles in the pathogenesis and/or complications of DM^12–14^. Some found EV involvement in atherosclerosis progression in DM^14^, others stated EV’s role as antigen presentation to the immune system or playing a messenger role between cells during inflammation^12,13^. Since we saw increased BBB permeability in type 1 and 2 animal models of DM, we hypothesized that this phenomenon might be attributed to the shedding of TJ proteins in EVs, thus affecting proper TJ presence in the endothelial membranes. To test this hypothesis, we isolated EVs from the serum of STZ-induced DM type 1 mice^3^ as described in Methods section. We used NanoSight’s nanoparticle tracking analysis (NTA) tool to characterize both EV size and concentration. The size of the EVs increased by 23% after 8 weeks since DM type induction by STZ-injection in mice, and 4 weeks later the size of the EVs in this group of mice was further boosted by 16% (Fig. 2a). Interestingly, we noticed that there was a significant rise of 1.4-fold in size of the EVs both in the control group of mice and STZ-treated mice when compared 8 and 12 weeks after the start of the experiment, while there was no further size increase when compared to STZ-treated group and control group at the 12-week time point (Fig. 2a). NTA revealed that upon development of DM there was a significant 1.6-1.8-fold increase in the amount of EVs in the serum (Fig. 2b). Isolated EVs were pulled-down utilizing CD63 Exo capturing magnetic beads, as described in methods, and stained for TJ proteins with anti-claudin-5 or occludin antibodies, and subjected to flow cytometry analysis. FACS analysis demonstrated that there was up to a 40% boost of occludin positive EVs in mouse serum at the 12-week time point (Fig. 2c), whereas there was substantial increase of claudin-5 positive EVs at both 8-week and 12- week time points (Fig. 2e). When analyzing protein expression levels on the EVs, we discovered that despite of difference in amount of occludin positive EVs only at 12-week time point, expression of occludin was significantly amplified at both 8- and 12-week points (Fig. 2d). Claudin-5 expression on EVs was increased by 1.28 ± 0.9-fold in mouse serum after 8 weeks after DM development that eventually decreased toward the end of the experiment (Fig. 2f). Elevated levels of expression of TJ proteins on EVs demonstrate another factor that might lead to increased BBB permeability due to the shedding of TJs from the endothelium.

DM conditions increase expression of adhesion molecules on BMVEC and subsequently lead to enhanced leukocyte adhesion to and migration across endothelial monolayer.

**Figure 3:**
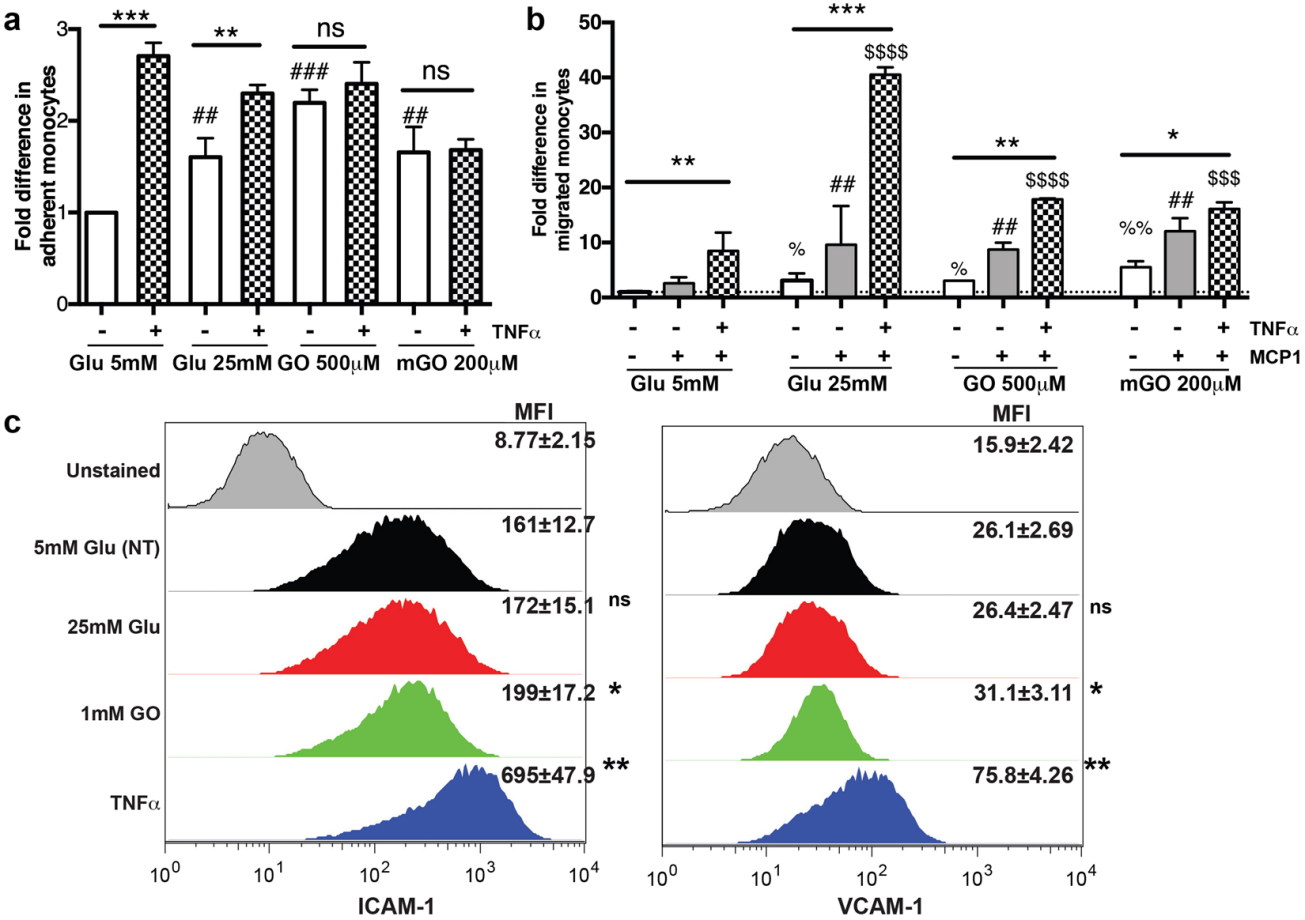
Hyperglycemia induces ICAM and VCAM expression in BMVEC and increases primary human monocyte adhesion to and migration across BMVEC monolayer. (**a**) BMVEC were exposed to TNFα (75 ng/mL, overnight) in conjunction with glucose (25 mM) or AGEs. Treatments were removed prior to addition of monocytes. Data are shown as fold difference (mean ± SEM) of adhesion, with adhesion to BMVEC alone assigned a value of 1. **p < 0.01 and ***p < 0.001 significance of TNFα vs control, ^##^p < 0.01 and ^###^p < 0.001 Glu 25 mM or AGEs vs. Glu 5 mM control (**b**) The migration assay was performed using inserts seeded with 2.5 × 10^4^ BMVEC/insert. MCP1 (30 ng/mL) was used as a relevant chemokine. BMVEC were exposed to TNFα and/or MCP1 in conjunction with glucose (25 mM) or AGEs. Monocytes were added to the upper chamber of inserts. Chemotaxis was allowed for 2 hr. Data are shown as fold difference (mean ± SEM) of migration, with migration of monocytes across BMVEC-only without TNFα and MCP1 assigned a value of 1. *p < 0.05, **p < 0.01 and ***p < 0.001 significance of TNFα vs control, %p < 0.05 and %%p < 0.01 Glu 25 mM or AGEs vs. Glu 5 mM without MCP-1/TNFα, ##p < 0.01 MCP1 plus Glu 25 mM or AGEs vs. Glu 5 mM control with MCP-1, but no TNFα, $$$p < 0.01 and $$$$p < 0.001 MCP1/ TNFα plus Glu 25 mM or AGEs vs. Glu 5 mM with MCP-1/TNFα. (**c**) Endothelial Cells were stimulated with 25 mM glucose or AGEs, then were labeled with fluorophore-labeled anti-ICAM or VCAM antibodies and expression was measured by FACS. Mean Fluorescent Intensity (MFI) from three independent experiments are shown as the mean ± SD. *p < 0.05 represent significance vs non-stimulated cells.

**Figure 4:**
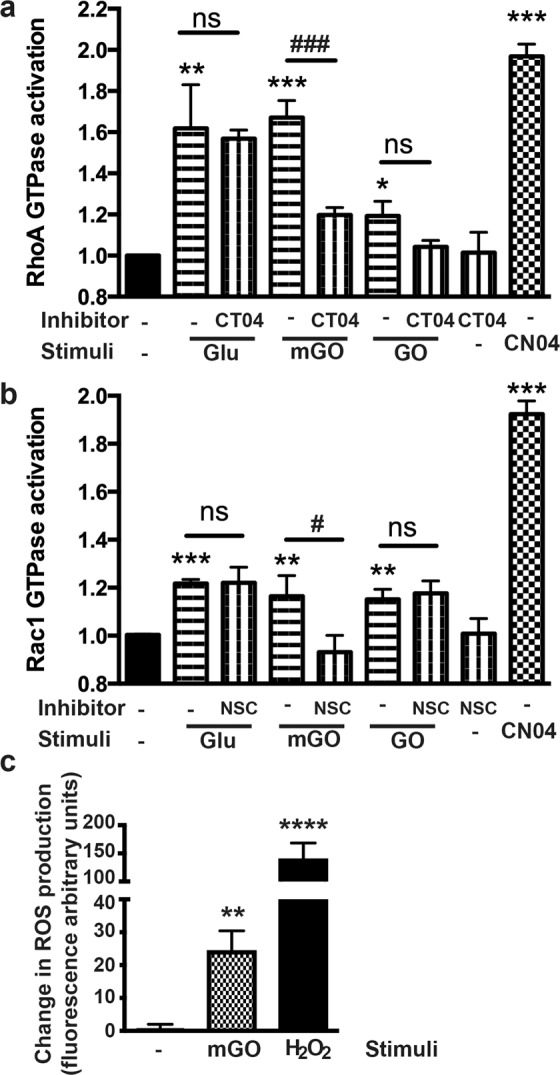
Hyperglycemia and AGEs activate small Rho GTPases in endothelial cells and increase ROS production. BMVEC were exposed to high glucose or AGEs. Rho A (a) and Rac 1 (b) GTPase activity was measured using G-LISA kits (Cytoskeleton Inc.). NSC and CT04 were used as Rac 1 and Rho A inhibitors, respectively, and CN04 was a positive control. c. ROS production was quantified in BMVEC. Results were normalized to baseline NT culture, and presented as ±SD from triplicate determinations. **p < 0.01 ***p < 0.005, ****p < 0.001 indicate significance vs. NT. ns, non-significant.

To mimic BBB injury in DM conditions *in vitro*, we modeled it in the previously established *in vitro* BBB model, primary human BMVEC^21,22,24,29,30^ from our laboratory using a combination of high glucose (HG) or AGEs. Several groups have shown increased levels of TNFα in blood of DM patients^31,32^. We treated BMVEC with TNFα under physiologic or hyperglycemic conditions and investigated whether these conditions would lead to altered leukocyte adhesion to and/or migration across the endothelial monolayer. Our results demonstrate that TNFα treatment led to a 2.7-fold increase in leukocyte adhesion to endothelium in physiological glucose concentration or 2.2-, 2.3- and 1.65-fold increases in HG, GO or mGO treatments, respectively (Fig. 3a). HG or AGEs escalated leukocyte adhesion even without TNFα addition to the media. A similar boost in leukocyte migration across the monolayer was noticed under DM conditions (Fig. 3b). Since we saw increased adhesion of primary monocytes to the brain endothelial monolayer, we decided to assess whether HG or AGEs would increase expression of adhesion molecules in BMVEC. Indeed, AGEs upregulated ICAM and VCAM expression (Fig. 3c), whereas HG did not affect levels of adhesion molecules. 1.6-fold in RhoA-GTP (Figs. 4a) and 1.21-fold in Rac1-GTP (Fig. 4b) in BMVEC stimulated with HG. Activity of both GTPases was augmented by AGEs treatment. Several groups demonstrated that hyperglycemic conditions and AGEs might result in oxidative stress responses^33,34^. Indeed, we found that AGEs treatment resulted in a significant increase of ROS production in endothelial cells (Fig. 4c).

**Figure 5:**
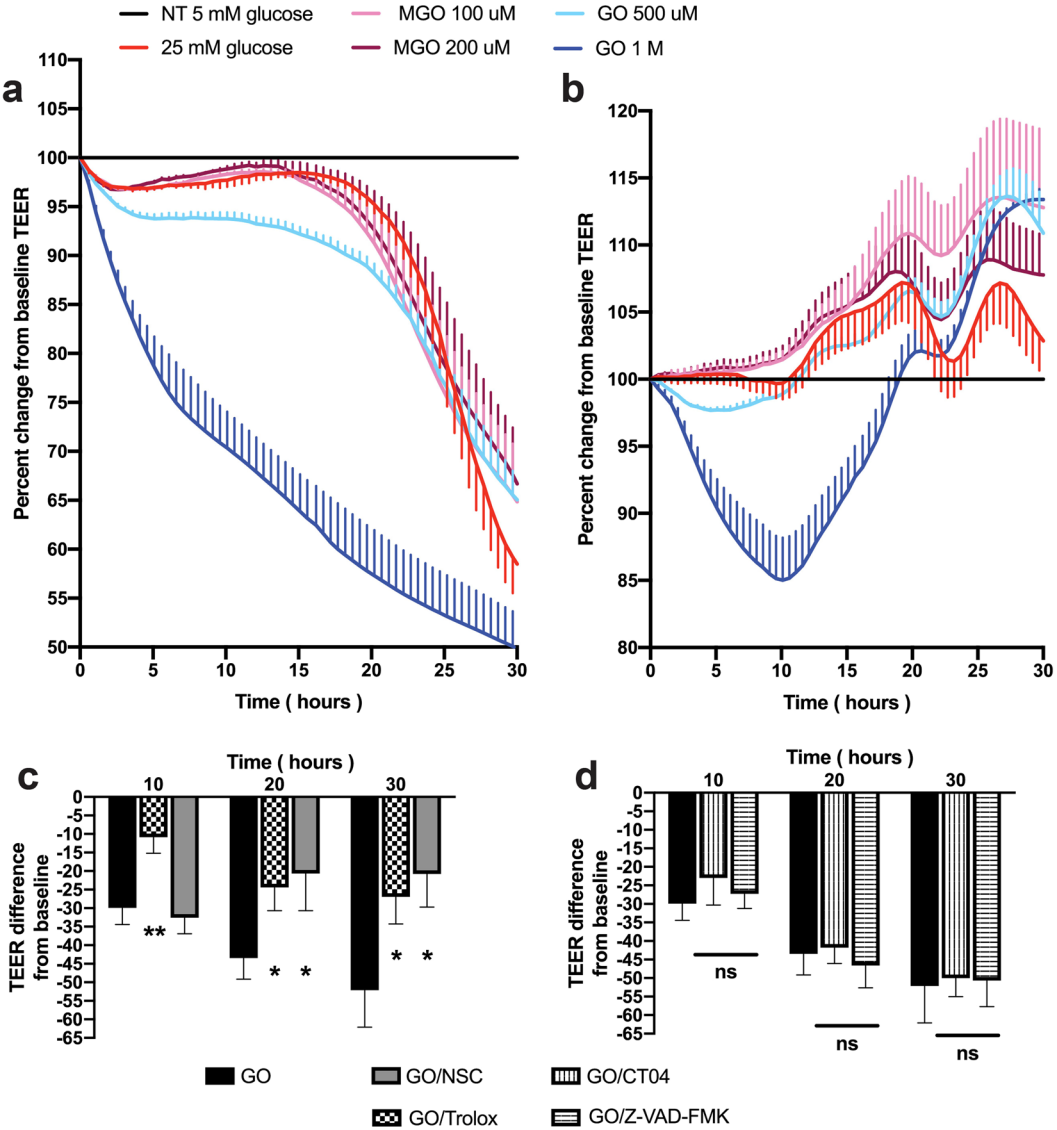
Hyperglycemia decreases tightness of the barrier. BMVEC alone (**a**) or in co-culture with pericytes (**b**) were exposed to 25 mM glucose or AGEs and transendothelial electric resistance (TEER) was acquired for a period of 30 hrs as described in methods. To inhibit RhoA or Rac1 GTPase activity, ROS or apoptosis, BMVEC were pretreated for 30 minutes prior to addition of AGE (GO) with specific inhibitors, 1 μg/ml CT04 (**d**), 75 μM NSC23766 (NSC) (**c**), 25 μM Trolox (**c**) or 100 μM Z-VAD-FMK (**d**), respectively. Results were normalized to baseline NT culture, and presented as ±SD from triplicate determinations. *p < 0.05 or **p < 0.01 indicate significance vs. NT. ns, non-significant.

To assess barrier function in diabetes, we measured TEER in BMVEC monolayers or their co-culture with pericytes. Exposure of BMVEC alone to HG resulted in an immediate drop of TEER and HG conditions showed further decline in BBB tightness (up to 40% of control after 24 h, Fig. 5a). Next, we investigated whether AGEs would have any influence on barrier function and found a dose-dependent effect with GO treatment (500 μM and 1 mM, light and dark blue lines), were higher concentration lead to 50% loss in TEER values. Interestingly, in BMVEC co-cultured with pericytes, only GO influenced barrier tightness in a dose-dependent manner. Neither HG nor mGO had any effect on two-cell BBB layer tightness (Fig. 5b). Next, we checked whether inhibition of small GTPases, ROS or apoptosis processes would rescue the loss in tightness caused by GO. Interestingly, Rac1 GTPase inhibitor (NSC) and ROS inhibitor (Trolox) were able to significantly alleviate GO-induced TEER drop (Fig. 5c). Neither RhoA small GTPase inhibitor (CT04) nor caspase inhibitor (Z-VAD-FMK) had any effect on BBB tightness decrease caused by GO (Fig. 5d). We performed viability test and did not see any cytotoxic effects (data not shown). Therefore, we conclude that hyperglycemic conditions lead to small GTPase activation and ROS production and potentially affect the actin cytoskeleton rearrangements and BBB tightness.

### Hyperglycemic conditions result in pericyte dysfunction

**Figure 6:**
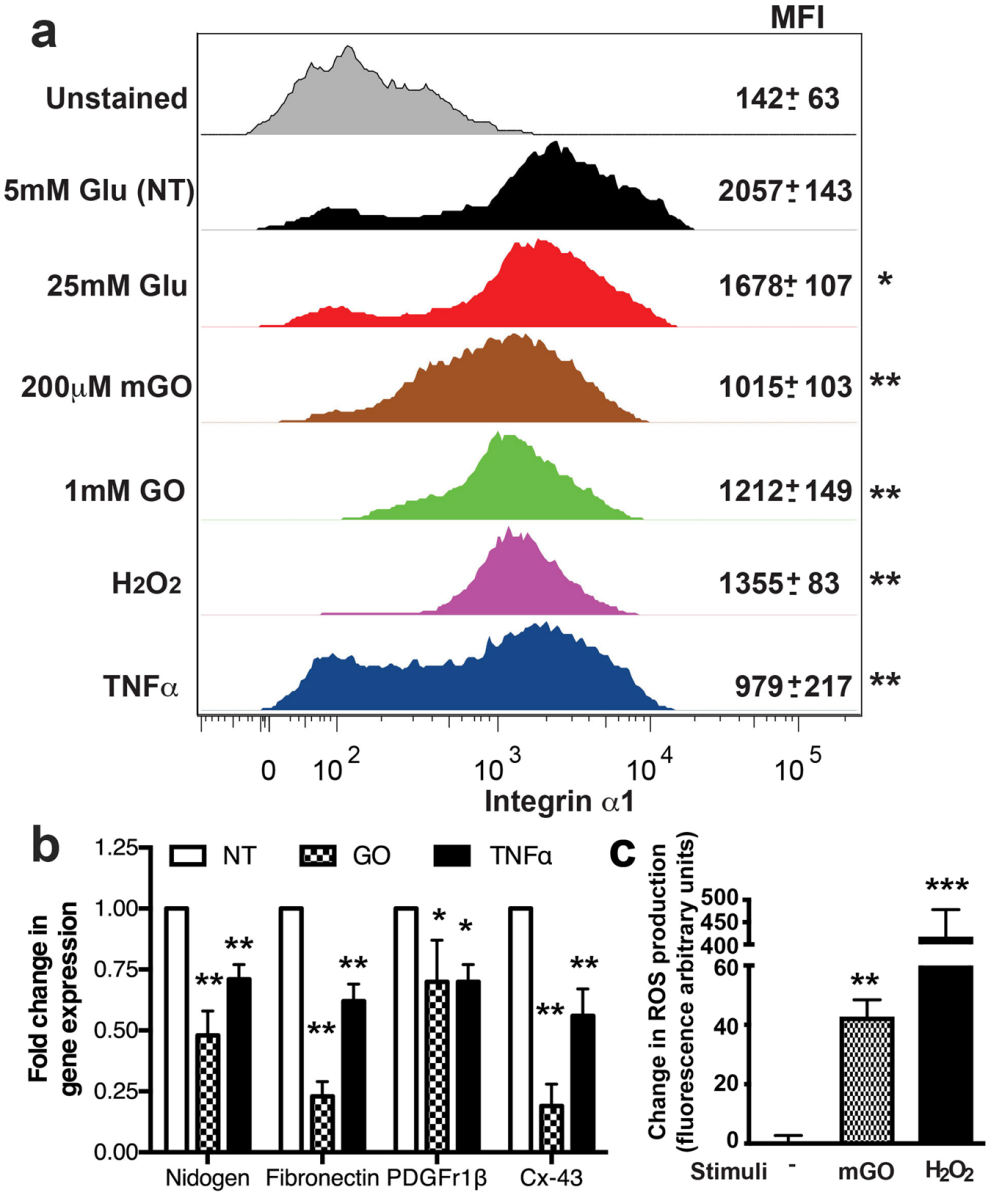
Hyperglycemia reduces expression of integrin α1 protein and genes of basement membrane components and increases ROS production in pericytes. Pericytes were stimulated with 25 mM Glu or AGEs, labeled with fluorophore-labeled anti-Integrin α1 Abs and expression was measured by FACS. (**a**) Representative histograms are shown for each treatment and Mean Fluorescent Intensity (MFI) from three independent experiments are shown as the mean ± SD. mRNA was isolated and quantified by qPCR. Fold change in gene expression from two independent experiments (performed in triplicate) is shown (**b**). (**c**) ROS production was quantified in pericytes. Results were normalized to baseline NT culture. *p < 0.05, **p < 0.01 or ***p < 0.001 represent significance vs. non-stimulated cells.

Lastly, we evaluated the effects of diabetic conditions on pericyte functions. We demonstrated that expression levels of integrin α1 (a key molecule assuring adhesion to basement membrane matrix) on pericytes was reduced by 19% (HG), whereas mGO and GO diminished it by 51% and 32%, respectively (Fig. 6A). Treatments with TNFα or H_2_O_2_ (to replicate inflammatory or oxidative stress conditions) diminished integrin α1 expression on pericytes as well. GO treatment led to a ~5.2-fold reduction in expression of gap junction protein Cx-43 (key for communication between BMVEC and pericytes). Similarly, exposure to AGEs resulted in a 20% ± 6% diminution in PDGF-Rβ expression (important for proper function of pericytes at the BBB). Basal membrane protein genes, fibronectin and nidogen, showed 4.1- and 2-fold downregulation, respectively, in pericytes challenged with GO (Fig. 6B). Next, we determined that AGEs treatment resulted in significant increase of ROS production in pericytes (Fig. 6C). In summary, using primary human brain endothelial cells and pericytes, we were able to demonstrate a pro-inflammatory phenotype and dysfunction of cellular elements of the neurovascular unit under diabetic conditions.

## Discussion

BBB dysfunction is often found in a wide range of VCID syndromes^35,36^. The BBB serves as a discriminating barrier preserving the homeostasis in the CNS by controlling ion balance, assisting in nutritional transport, and blocking influx of potentially neurotoxic molecules from the circulation at the level of the cerebral microvascular endothelium^35,36^. DM is a metabolic disorder depicted by hyperglycemia leading to end-organ injury in different organs due to microvascular compromise (cardiovascular disease, nephropathy and retinopathy) and inflammation. Learning disabilities and memory deficits have been documented in DM type 1 and type 2 patients^37–39^, which might be due to cerebral vascular dysfunction. Connection of microvascular changes to cognitive deterioration in DM has not been substantiated until very recently, with defects noted in neuronal function, metabolic function, white matter microstructure, and blood perfusion^40,41^.

It has been implied in the literature, that BBB breakdown as one of the grounds of dementia in DM and AD^28^. However, the precise mechanisms of injury, association amongst enhanced permeability and memory loss, DM and therapeutic potential of BBB protective approaches in cognitive decline are presently unknown. Here, we scrutinized the idea that DM settings decrease BBB integrity directly (via effects on brain endothelium and pericytes, separately and together) and stimulate a pro-inflammatory phenotype of brain endothelium (resulting in a low-level inflammation) that further aggravates harm of the barrier. We postulated that BBB integrity might be affected in DM due to inopportune folding or incorporation in cell membranes. In the current study, we checked this hypothesis by assessing TJ localization (membrane incorporation) in BMVs which were subjected to DM conditions *ex vivo*. Treatments with AGEs, such as GO, caused significant reduction of both claudin-5 and occludin protein expression, while MGO treatment resulted in significant diminution of claudin-5 in TJ staining. Similar reduction in TJ expression for both occludin and claudin-5 were noticed in LPS *ex vivo*-treated BMVs. These results suggest the commonality of the hyperglycemic DM environment and pro-inflammatory conditions, in this case caused by LPS.

In recent years, there have been major advances in the understanding of the biology of extracellular vesicles. Clinical and experimental studies have shown that EVs play a vital role in the pathophysiology of inflammation-associated disorders. A cardinal feature of these disorders is an enhanced generation of platelet-, endothelial-, and leukocyte-derived EVs^42^. Here in our study we determined that hyperglycemic conditions led to a significant increase of EVs in serum of DM type 1 mice. Interestingly, DM progression also led to an increase in size of the EVs, from 110 ± 12 nm to 138 ± 15.3 nm. Microvesicles are generally larger than exosomes (100–1000 nm vs. 30-120 nm, respectively). Microvesicles are formed by direct budding of the plasma membrane, whereas exosomes are formed within endosomal compartments and secreted by the fusion of multivesicular bodies with the plasma membrane. Apoptotic bodies are released upon programmed cell death by membrane blebbing and can be from 50 nm to 5 μm in diameter. However, due to a significant overlap in size, similarities in composition and lack of specific markers, it is very difficult to assign individual EVs to one of the biogenesis pathways. Recently, small GTPases were shown to contribute EV production and secretion^43,44^. Further studies are required to determine what type of EVs were found in our study, and which mechanism was involved. Additionally, EVs isolated from serum from DM type 1 mice had a significantly high content of the endothelial TJ proteins, claudin-5 and occludin compared to normal glycemic mice. Presence of the TJ on EVs could potentially explain why the BBB in DM mice was compromised and why these mice shown higher significant reduction in TJ occludin and claudin-5 protein expression in immunohistochemical examination^3^ and ZO-1 protein (Supplemental Fig. 3). Previously we also identified that at least occludin mRNA levels were highly increased in microvessels isolated from DM mice, implying either a compensatory phenomenon in DM to compensate leakage of the TJ protein via EVs or improper incorporation in cell membranes. Further studies will be required to test whether ZO-1 protein is shed by EVs or its expression is affected on transcriptional/translational levels. Additionally, presence of the TJ proteins on the EVs might serve as a potential biomarker of BBB damage. Further studies are required to see whether TJ presence on EVs would correlate with dementia progression.

To simulate BBB injury in DM conditions *in vitro*, we used the previously established in our lab *in vitro* BBB model, primary human BMVEC^21,22,24,29,30^ using combination of HG or AGEs. Since some research groups^31,32^ have shown increased levels of TNFα in blood of DM patients, we treated BMVEC with TNFα under normal or hyperglycemic conditions and investigated to see whether these conditions would lead to altered leukocyte adhesion to and/or migration across the endothelial monolayer. Indeed, our results demonstrate that TNFα treatment led to an increase in leukocyte adhesion to endothelium under physiological glucose concentration and hyperglycemic conditions. HG or AGEs caused enhanced leukocyte adhesion even without TNFα addition to the media. A boost in leukocyte migration across the monolayer was noticed under DM conditions. Because we saw increased adhesion of primary monocytes to the brain endothelial monolayer, we decided to evaluate whether HG or AGEs would increase expression of adhesion molecules in BMVEC. Indeed, AGEs upregulated ICAM and VCAM expression, whereas HG did not affect adhesion molecule expression levels. Altannavch and colleagues^45^ have shown detectable effects of HG on the adhesion molecules (VCAM and ICAM) and TNFα cytokine expression in human umbilical vein endothelial cells (HUVEC) after 24 hours. Another group shown that longer exposure to HG is required for microvascular endothelial cells isolated from bovine or aortic aortas or skin to elevate VCAM expression^46^. Others demonstrated that intermittent exposure of HUVEC to HG also elevates adhesion molecule expression^47^, which can be explained by the difference in response of endothelial cells of different origin.

A critical role in determining junctional integrity between endothelial cells and permeability regulation of the endothelium is assigned to the actin cytoskeleton machinery^16,48,49^. The microvascular endothelium controls discriminatory permeability of the BBB to fluids and solutes. Since small GTPases, RhoA and Rac1, control cytoskeleton, TJ and adhesion molecule expression in BMVEC and endothelial cells of other organs^15–17^, we examined the ability of DM conditions to affect their activation. Indeed, both RhoA and Rac1 GTPases show a significant increase in their activation in BMVEC stimulated with HG. Activity of both GTPases was augmented by AGE treatments as well. Exploiting our BBB *in vitro* model, we plated BMVEC alone or in co-culture with pericytes and measured endothelial barrier function utilizing TEER. Exposure of BMVEC alone to HG resulted in immediate drop of TEER and HG conditions showed further decline in BBB tightness, and exposure to AGEs (GO) resulted in a dose-dependent effect the endothelial barrier function. Interestingly, when BMVEC were co-cultured with pericytes, only GO influenced barrier tightness (also in a dose-dependent manner); however, neither HG nor mGO had any effect on two-cell BBB layer tightness. Intriguingly, Rac1 GTPase inhibitor (NSC) and ROS inhibitor (Trolox) were able to significantly improve GO-induced TEER decline. However, neither RhoA small GTPase inhibitor (CT04) nor caspase inhibitor (Z-VAD-FMK) had any effect on GO-caused BBB layer tightness diminution Therefore, we conclude that hyperglycemic conditions lead to small GTPase activation and ROS production and potentially affect the actin cytoskeleton rearrangements and BBB tightness.

Lastly, we evaluated the effects of diabetic conditions on pericyte functions. We and others have shown that integrin α1 levels are affected in different pro-inflammatory conditions^10,25,35,36^. HG and AGEs exposure resulted in significant reduction of integrin α1 protein level. Treatment with TNFα or H_2_O_2_ (to replicate inflammatory or oxidative stress conditions) diminished integrin α1 expression on pericytes as well. Since integrin α1 is a key molecule assuring adhesion to the basement membrane (BM) matrix on pericytes, such alteration may lead to defective attachment to BM, impaired microvessel stability and hemorrhage^50^. Down-regulation of BM protein mRNAs, such as fibronectin and nidogen, were found to be down-regulated in hyperglycemic conditions in pericytes. Pericytes, along with brain endothelium, synthesize the BM and its abnormalities are known to be present in a number of neurodegenerative and neurovascular diseases^18^. Hyperglycemic conditions resulted in significant reduction in mRNA levels of PDGF-R1β and Cx-43 which are important for pericyte function^25,51–53^. Our results indicate that both BMVEC and pericytes produce augmented amounts of ROS. Oxidative stress in hyperglycemic conditions could be very harmful for proper endothelial cell and/or pericyte function^33,34,54^. In summary, using primary human brain endothelial cells and pericytes, we were able to demonstrate a pro-inflammatory phenotype and dysfunction of cellular elements of the neurovascular unit under diabetic conditions.

## Supplementary information

Supplementary Information.
