# Author Correction: Hyperglycemia and advanced glycation end products disrupt BBB and promote occludin and claudin-5 protein secretion on extracellular microvesicles

**DOI:** 10.1038/s41598-020-75940-7

**Published:** 2020-10-28

**Authors:** Slava Rom, Nathan A. Heldt, Sachin Gajghate, Alecia Seliga, Nancy L. Reichenbach, Yuri Persidsky

**Affiliations:** 1 Department of Pathology and Laboratory Medicine, Lewis Katz School of Medicine, Temple University, Philadelphia, PA 19140 USA; 2 Center for Substance Abuse Research, Lewis Katz School of Medicine, Temple University, Philadelphia, PA 19140 USA

Correction to: *Scientific Reports* 10.1038/s41598-020-64349-x, Published online 29 April 2020

This Article contains an error in the Discussion section.

“Additionally, presence of the TJ proteins on the EVs might serve as a potential biomarker of BBB damage. Further studies are required to see whether TJ presence on EVs would correlate with dementia progression.”

should read:

“Additionally, presence of the TJ proteins on the EVs might serve as a potential biomarker of BBB damage. Presence of TJ protein, claudin-5, on EVs has been previously described in mice during neuroinflammation after traumatic brain injury (TBI) and in experimental autoimmune encephalomyelitis (EAE) model^1,2^. Further studies are required to see whether TJ presence on EVs would correlate with dementia progression.”

The omitted references are provided below.
