# Supplementary material for: Hyperglycemia and advanced glycation end products disrupt BBB and promote occludin and claudin-5 protein secretion on extracellular microvesicles
Source: Sci Rep. 2020 Apr 29;10:7274. doi: 10.1038/s41598-020-64349-x (PMC7190636; doi:10.1038/s41598-020-64349-x)

**Supplemental Figures for paper "Hyperglycemia and advanced glycation end products disrupt tight junction complexes and promote occludin and claudin-5 protein secretion on extracellular microvesicles"**

Slava Rom[1,2], Nathan A. Heldt[1], Sachin Gajghate[1], Alecia Seliga[1], Nancy L. Reichenbach[1] and Yuri Persidsky[1,2]

*[1]Department of Pathology and Laboratory Medicine, [2]Center for Substance Abuse Research, Lewis Katz School of Medicine, Temple University, Philadelphia, PA 19140, USA.*

**Supplemental Figure 1**. **Schematic representation of the image analysis process which was applied to quantify localization of claudin-5 or occludin.** Boxed text indicates modules ran in NIS-Elements General Analysis 3 package, with boldface indicating the module name and bulleted text indicating the corresponding parameters. Arrowhead indicates an instance of immunostaining outside the vessel volume which was eliminated during analysis by application of the vessel mask. Analysis software provided quantification of the indicated parameters which were used to calculate a localization ratio.

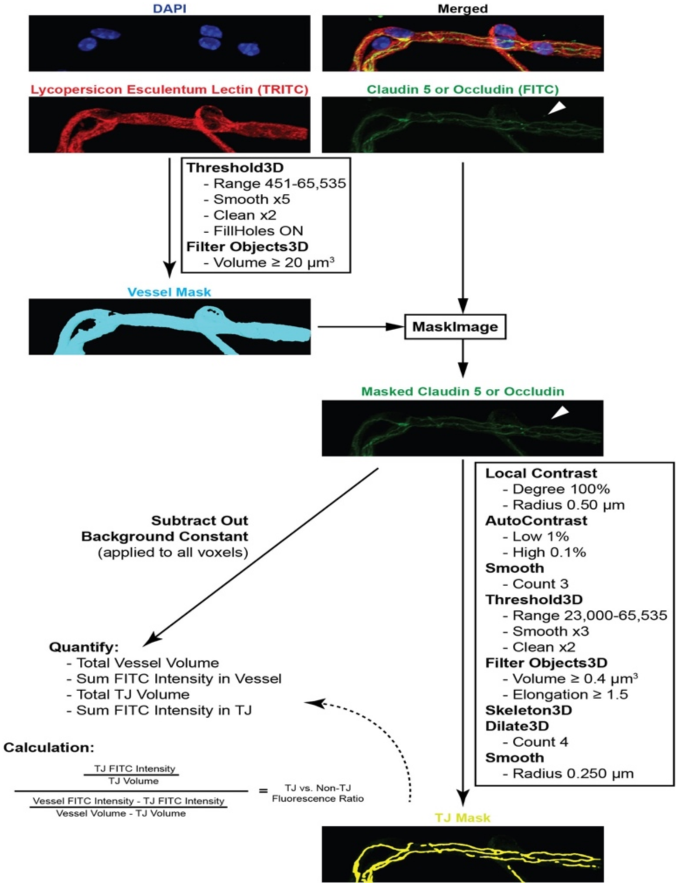

**Supplemental Figure 2. AGEs disrupt TJ complexes in microvessels treated *ex vivo*.** Isolated vessels were labeled with *Lycopersicon esculentum* lectin (LEL, red), DAPI (blue), and immunostained for either claudin-5 or occludin. Multiple vessels ($n$ = 7-8) were imaged from each treatment group and the total mean fluorescent intensity for claudin-5 (a, c and e) or occludin (b, d and f) is shown for junctional and non-junctional localization was quantified as described in methods. Filled circles indicate the data point corresponding to the representative image shown in Figure 1 (main manuscript). Scale bar 10 µm, *$p$ = 0.05 versus NT. NT, no treatment; LPS, lipopolysaccharide; GO, glyoxal; MGO, methylglyoxal; HG, high glucose.

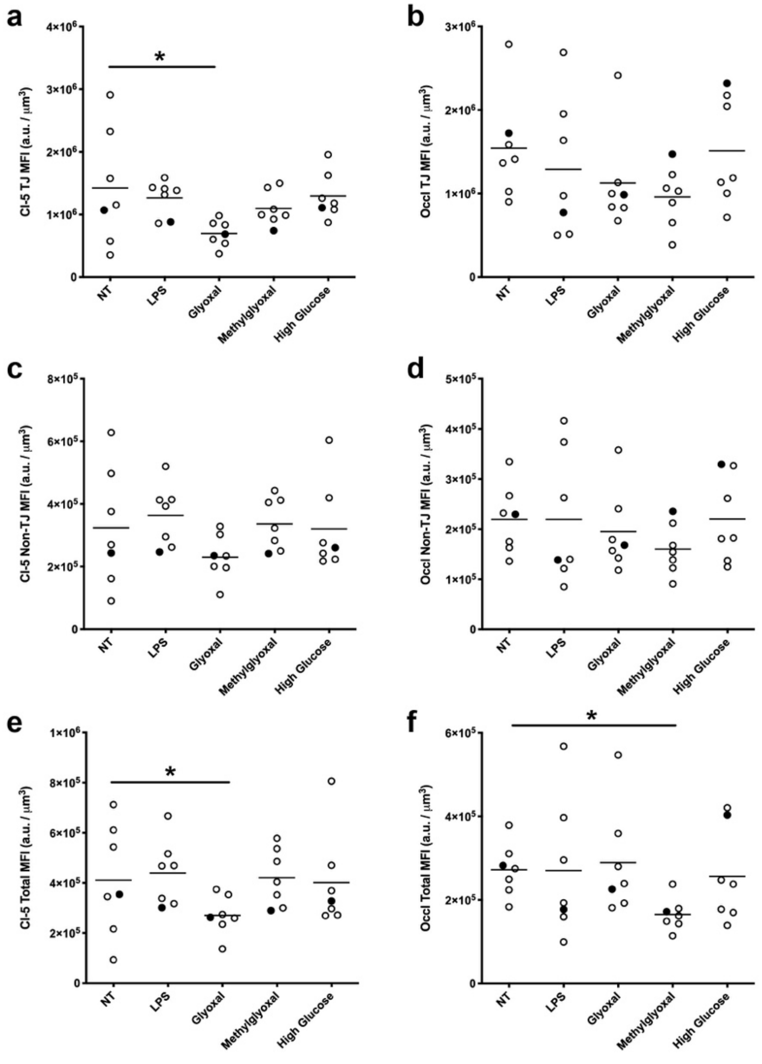

**Supplemental Figure 3. ZO-1 protein expression is decreased in BMVs in DM type 1 and 2 models.** ZO-1 immunostaining is diminished (ZO-1, brown) in db/db (upper row) or STZ-treated (lower row) vs. respective control mice (a). Original magnification was x 400. Semi-quantitative evaluation of ZO-1 (b) staining was performed as described[3].

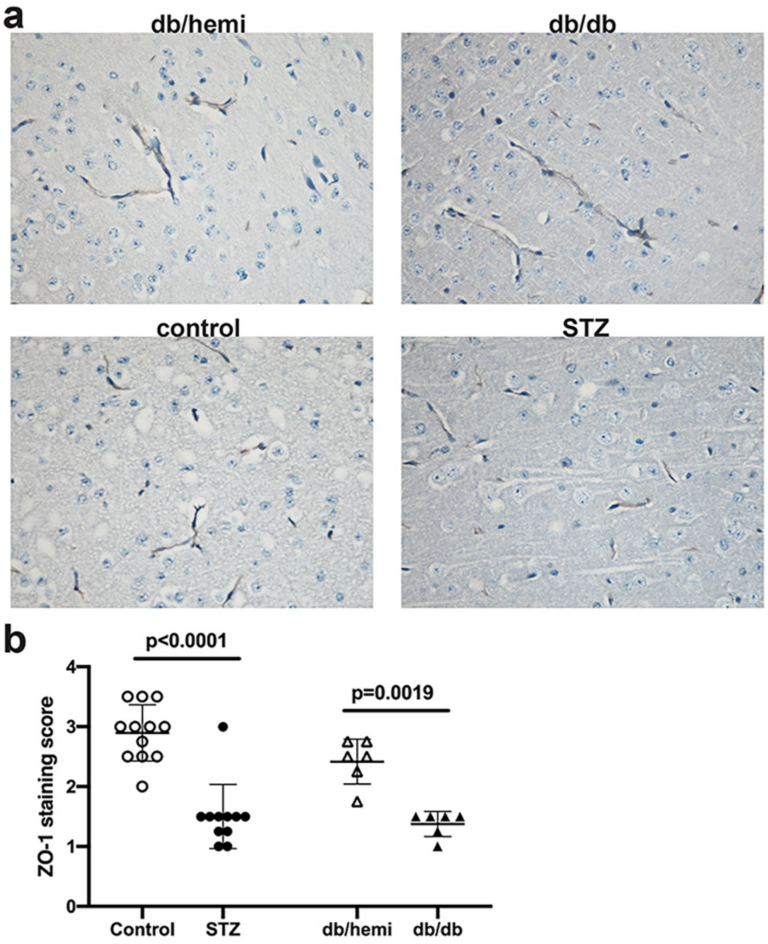

Supplement: Supplementary file 1 — Supplementary Information. [file 41598_2020_64349_MOESM1_ESM.pdf]
